# Electron-beam lithography of cinnamate polythiophene films: conductive nanorods for electronic applications†

**DOI:** 10.1039/d2sc01867e

**Published:** 2022-06-16

**Authors:** N. Maximilian Bojanowski, Christian Huck, Lisa Veith, Karl-Philipp Strunk, Rainer Bäuerle, Christian Melzer, Jan Freudenberg, Irene Wacker, Rasmus R. Schröder, Petra Tegeder, Uwe H. F. Bunz

**Affiliations:** Organisch-Chemisches Institut, Ruprecht-Karls-Universität Heidelberg Im Neuenheimer Feld 270 69120 Heidelberg Germany uwe.bunz@uni-heidelberg.de; Centre for Advanced Materials, Ruprecht-Karls-Universität Heidelberg Im Neuenheimer Feld 225 69120 Heidelberg Germany; Physikalisch-Chemisches Institut, Ruprecht-Karls-Universität Heidelberg Im Neuenheimer Feld 253 69120 Heidelberg Germany; InnovationLab GmbH Speyerer Straße 4 69115 Heidelberg Germany; BioQuant, Ruprecht-Karls-Universität Heidelberg Im Neuenheimer Feld 267 69120 Heidelberg Germany

## Abstract

We report the electron-beam induced crosslinking of cinnamate-substituted polythiophene proceeding *via* excited state [2+2]-cycloaddition. Network formation in thin films is evidenced by infrared spectroscopy and film retention experiments. For the polymer studied herin, the electron-stimulated process appears to be superior to photo (UV)-induced crosslinking as it leads to less degradation. Electron beam lithography (EBL) patterns cinnamate-substituted polythiophene thin films on the nanoscale with a resolution of around 100 nm. As a proof of concept, we fabricated nanoscale organic transistors using doped and cross-linked P3ZT as contact fingers in thin film transistors.

## Abstract 2

Electron beam lithography patterns selectively cinnamate-substituted polythiophene thin films *via* [2+2]-cycloaddition. A nanoscale organic field effect transistor is constructed using cross-linked and doped polythiophene as electrodes.

## Graphical abstract

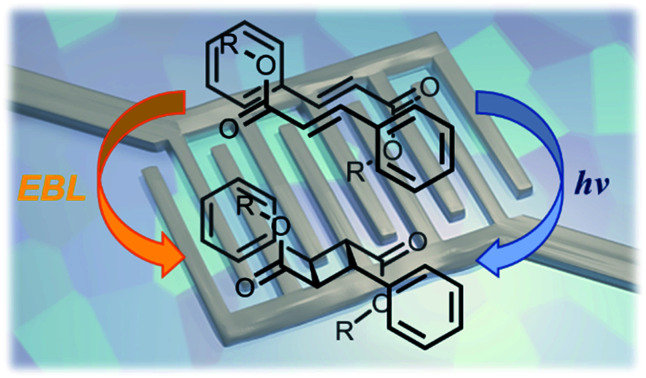

## Introduction

Manufacturing multi-layer electronic components requires control of size and structure of functional layers such as electrodes, semiconductors, and dielectrics.^1^ Light patterns photoactive materials, either through shadow masks or with focused beams (direct laser writing, DLW) on a submicron scale.^2,3^ Photons desolubilize the film, changing its chemical and/or physical properties to allow film development, which removes unreacted starting materials revealing the desired photopattern.

**Fig. 1:**
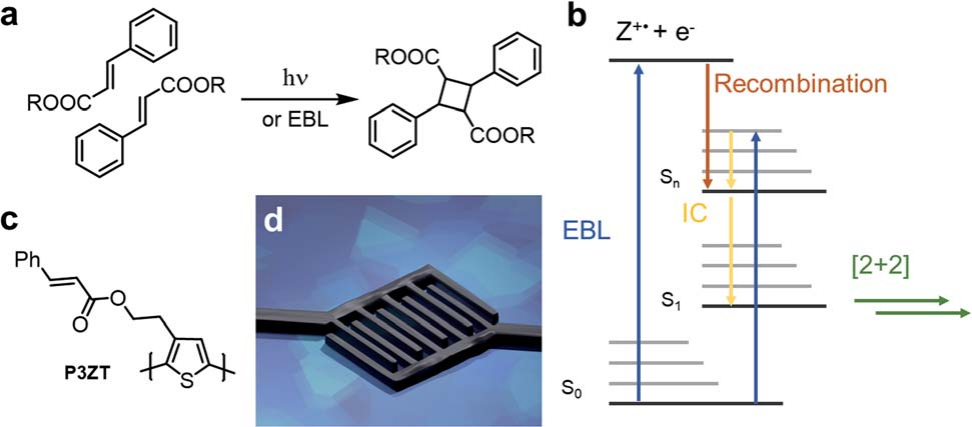
(a) Dimerization of cinnamic esters to truxillic acid esters induced by photons or electrons (electron beam lithography: EBL). (b) Proposed mechanism of the EB-induced [2+2] cycloaddition of cinnamic esters. (c) Structure of cinnamic ester-substituted P3ZT. (d) Illustration of the application of doped and immobilized polythiophene -cinnamicester nanorods as contact material in nanotransistors.

Cinnamates react photochemically in a Woodward–Hoffmann allowed [2+2]-cycloaddition yielding cyclobutanes (Fig. 1a).^4,5^ This photodimerization cures electro-woven fibers, stabilizes self-healing polymer networks for artificial tissues, and patterns substituted nanoparticles in 2D microelectronic devices.^6–8^ A disadvantage of such a single-photon process is the restricted resolution (>200 nm) due to the diffraction limit in patterning applications. Direct laser writing (DLW) usually relies on a two-photon process to achieve high resolution and therefore needs high laser intensities at the writing focus. Thus, a high optical transparency of the materials for DLW is required. This excludes DLW for most organic semiconductors, as these are inherently coloured due to their charge transporting π-electron systems. In contrast, the high resolution of electron-beam lithography (EBL) offers opportunities for photo processed, miniaturized devices.

When irradiated with electrons, organic thin films crosslink and undergo a change in local solubility.^9,10^ Persson *et al.* structured poly(3-octylthiophene) with EBL and doped the resulting structures with iron(iii) chloride.^11^ Hikmet *et al.* patterned poly(*para*-phenylenevinylene)-derivatives (PPV) for multicolour organic light emitting diodes (OLEDs).^9^ In both cases, solubility reduction, necessary for solution processing of multi-layered organic electronic devices, avoids the intermixing of consecutive layers.^12–21^ Although resolution beyond the diffraction limit of light is possible, these electron-induced processes crosslink the polymers non-specifically through generated radicals.

Electron bombardment also induces reactivity known from photochemistry: electrons induce the *E*/*Z* isomerization of *cis*-cinnamic acid and its dimerization to truxillic acid in a cooled single crystal of *trans*-cinnamic acid (Fig. 1a).^22,23^ Recently, we “photo”-crosslinked a tetracinnamate-based monomer by high-energy electron radiation (10 keV) triggering the [2+2] cycloaddition.^24^ Robust nanostructures (resolution 60 nm) resist wet-chemical etching. The electron beam induced crosslinking is mechanistically similar to that of photoreactions of cinnamates,^23,24^ as summarized in Fig. 1b.

After electron impact, the cinnamate is directly excited to a higher excited state (S_2,3…*n*_), or such a state is populated by reaction of the radical cation (Z^+^) with a free electron (e^−^) – recombination occurs within picoseconds.^23^ After internal conversion to the first excited state (S_1_), the cinnamates dimerize in a fashion akin to light-induced cycloadditions. Excited singlet and triplet states of cinnamic acid derivatives undergo EB-induced cycloaddition.^22,23^

In this study, we use EBL to achieve high-resolution patterning of the cinnamate-functionalized polythiophene P3ZT (Fig. 1c). Nanostructuring of spin-coated thin films was achieved by EBL *via* [2+2] cycloaddition as evidenced by infrared (IR) spectroscopy. We created 110 nm thin features after developing. Solution based doping with iron(iii) chloride resulted in conductive nanowires, used as electrodes (Fig. 1d) in organic field-effect transistors (OFETs) with 6,13-bis(triisopropylsilyl)pentacene TIPS-Pen (ref. 25) as a hole transporter.

## Results and discussion

### Synthesis and properties

**Scheme 1:**
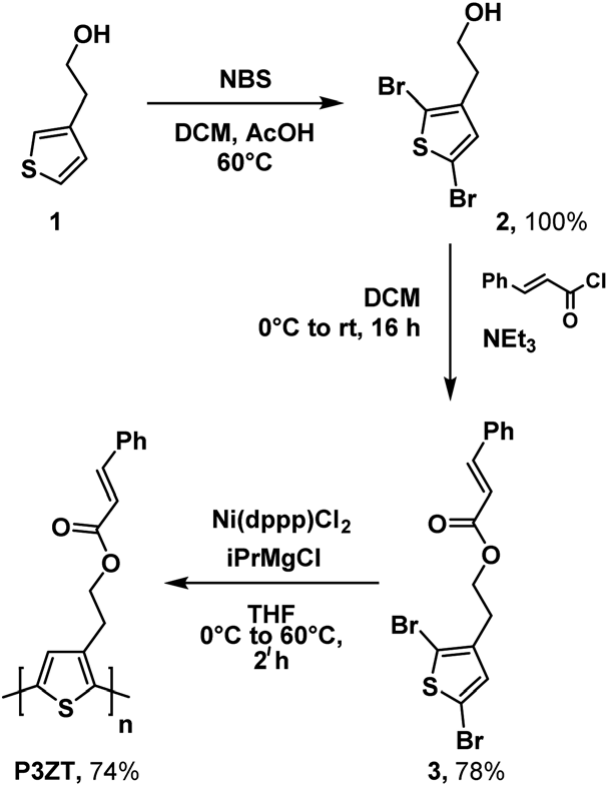
Synthesis of P3ZT*via* Grignard metathesis.

2-(Thiophen-3-yl)ethan-1-ol 1 and *N*-bromosuccinimide (NBS) gave dibromothiophene 2; reaction with cinnamic acid chloride to furnished monomer 3 (Scheme 1, 78% yield over 2 steps).

Polymerization of 3 into the regioregular polythiophene P3ZT was carried out according to a modified preparation based on Grignard metathesis (GRIM) with Ni(dppp)Cl_2_ as catalyst. Similar to previous reports, the ester groups tolerated the Grignard reagent.^26^ After purification by Soxhlet extraction, P3ZT was obtained in 74% as a dark purple solid with metallic luster (ESI, Fig. S1†). Analytical gel permeation chromatography (GPC) *vs.* polystyrene determined the mean chain length to 74 repeat units (*M*_w_ = 1.9 × 10^4^ g mol^−1^), the head-to-tail (HT) content is 95% according to ^1^H NMR analysis.^27^P3ZT is stable up to 300 °C (see ESI, Fig. S2† for TGA/DSC). The optical and electronic properties in thin films are summarized in Table 1. Similar to P3HT (93% HT, *M*_w_ = 2.4 × 10^4^ g mol^−1^), thin film spectra of P3ZT exhibit two main absorption bands in the UV-vis at *λ* = 500 and 560 nm (P3HT: 546 and 602 nm) with a shoulder at 610 nm (see ESI, Fig. S4†). The absorption onsets of both polymers are similar (∼1.9 eV), the maxima at short wavelengths are blue-shifted by ∼60 nm, most likely a consequence of reduced planarization due to the cinnamyl substitution.^28^ On the other hand, intermolecular interaction between adjacent cinnamate units and polymer backbone can blue-shift the absorption spectra,^29^ as indicated by a broader absorption band (Fig. S4†).

**Table 1:** Optical and electronic properties of P3ZT and P3HT as a reference in thin films (*E*_g,opt._ = optical band gap, IP = ionization potential, EA = electron affinity, WF = work function)

Polymer	*λ*_onset_ [nm]	*E*_g,opt._ [eV]	IP^a^ [eV]	EA^b^ [eV]	WF [eV]
P3ZT	660	1.88	4.8	−3.0	4.0
P3HT	652	2.0	5.2^c^	−3.2^c^	4.8^30^

a Cyclovoltammetry (CV) measured in THF (with *n*-butylammonium hexafluorophosphate as electrolyte and ferrocene as external standard).

b EA was calculated from IP and *E*_g,opt_.

c Manufacturer information.

P3ZT is weakly fluorescent in solution (*λ*_max_ = 568 nm) and non-fluorescent in thin films from chloroform (10 mg mL^−1^) on silicon wafers with thermally grown silicon oxide (100 nm). X-ray photoelectron spectroscopy (XPS) analysis (ESI, Fig. S18†) supports the purity of the obtained material. Signals originating from catalyst residues or magnesium salts were absent. The traces of bromine originate from the one-sided chain growth of GRIM. The work function (WF) amounted to 4.0 eV (ESI, Fig. S19†).

### Electron irradiation

**Fig. 2:**
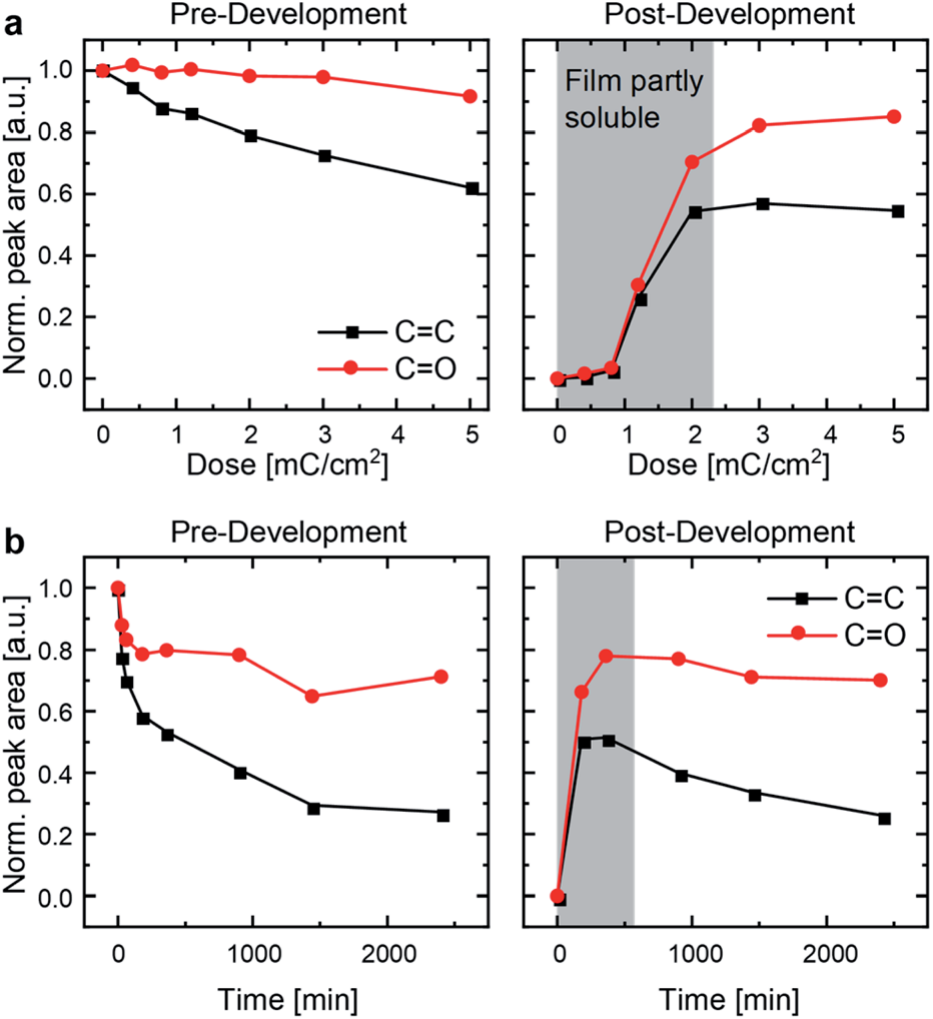
Normalized peak areas of the C=O peak (1675–1800 cm^−1^) and the C=C peak (1615–1670 cm^−1^) of IR spectra as a measure of chemical-bond type occurrence for (a) EBL and (b) UV-crosslinked P3ZT.

The influence of high-energy electrons (15 keV) on thin films of P3ZT was investigated by IR spectroscopy. Apart from the change of the IR fingerprint region, the decrease of the C=C vibrational stretching band at *ν*_C=C_ = 1635 cm^−1^ as well as at *ν*_C=CH_ at 3082 cm^−1^ (C–H stretching band) indicates [2+2] cycloaddition (Fig. 2 and S7†).^24,31^ IR spectra of UV-immobilized films (Fig. S6†) are superimposable to those obtained after EB treatment, indicating similar or identical reactivity under both types of irradiation. P3ZT is crosslinked and immobilized – it forms a 3D network. Non-crosslinked polymer films were dissolved by development in chloroform for 1 min. At low EB doses (<2.2 mC cm^−2^) or UV irradiation periods (<900 min), the films were partially dissolved after development, *e.g.* top layers were washed off. After sufficient exposure (≥3 mC cm^−2^), the IR signal intensity remained constant, indicating only minor material loss.

The integrated IR signals illuminate the solid-state reactivity. For both methods, the decrease in C=C peak areas were much more pronounced than that of the C=O peak, supporting the cycloaddition between double bonds. Without by-products or decomposition, one would not expect a significant reduction of C=O peak intensity. Surprisingly, we observe a strong decrease during the initial period of UV irradiation insufficient for effective crosslinking. Carbonyl peak intensities decrease to 80% (EBL) and 70% (UV-crosslinking) for insoluble and undeveloped thin films alike. After development, both the C=O and C=C peak areas are, as expected, invariant for EBL desolubilized films (Fig. 2a, right), whereas they continue to decrease with increasing irradiation time under UV conditions (Fig. 2b, right). Both observations indicate EBL-induced reactions to be at least on par to photoirradiation.

The film retention curves describing dose-dependent film thicknesses after development (ESI, Fig. S8†) indicate an inverse dependence of the required dose with the structure size because of scattering. Structures with a lateral diameter of >500 nm appear at a dose of 1.5 mC cm^−2^. Finer structural motifs require doses > 2.0 mC cm^−2^. This is rationalized with the proximity effect.^32^ Electrons that were focused to expose a particular structure over-expose the intended area into adjacent regions. This leads to an enlargement of the written structures. In contrast to small molecules previously reported, a lower cross-linking degree suffices to induce polymer immobilization through network formation.^24^ Surface charging additionally limits the resolution. 70 nm is achievable at a dose of 2.5 mC cm^−2^ (ESI, Fig. S9B†). For EBL, the writing process for the USAF resolution chart (group 10–15) takes 75 seconds. Crosslinking by UV irradiation requires >5 h in our experimental setup (with more concomitant radiation damage, see Fig. 2) as a consequence of the competition between absorption by the conjugated system and local excitation of the cinnamic acid moiety inducing crosslinking.

**Fig. 3:**
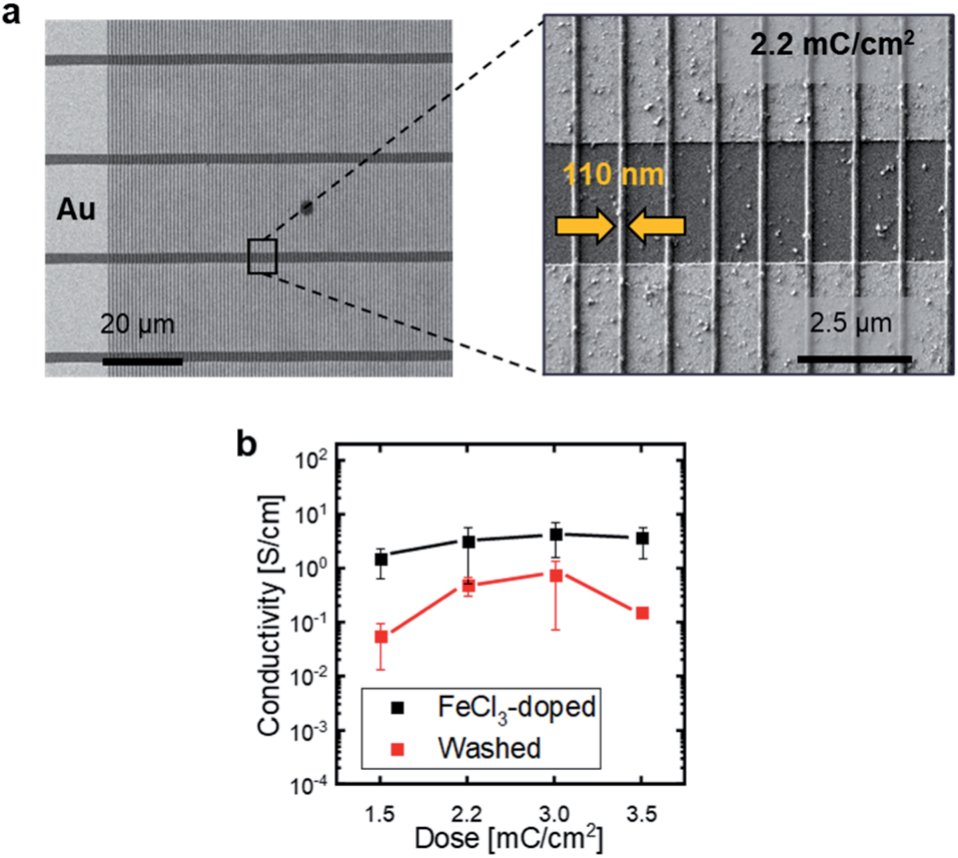
(a) EBL nanostructured P3ZT on pre-patterned end-of-line substrates. Overview image of parallel lines across multiple gold contacts and magnified section of individual lines written by a dose of 2.2 mC cm^−2^. (b) Average conductivity of P3ZT doped with FeCl_3_ before and after washing and the dependence of conductivity on EBL dose.

To assess the homogeneity of the immobilized nanostructures, films of P3ZT were EBL-patterned into 100 nm diameter, half-cylindrical nanowires and developed in chloroform. At a dose of 800 μC cm^−2^, inhomogeneous (wobbly) structures with washed-out resolution result (ESI, Fig. S11†). At 2.2 mC cm^−2^, defect-free straight lines with a width of about 110 nm are observed (Fig. 3). The defect free wires' effective width is 110 nm. With further increased dose, radiation damage appears in the material, dividing the nanowires into segments with different contrast and work function (ESI, Fig. S11†).

### Device fabrication

Next we doped the wires^33–36^ – conductive nanowires are useful in microelectronics on flexible substrates and for nanophotonics and plasmonics.^11,37–39^

Doping of pristine spin-cast films of P3ZT occurs by spin-coating of an iron(iii) chloride solution in DMF (1 mg mL^−1^) on top and subsequent washing of the films with acetonitrile to remove the excess of FeCl_3_. Doping renders the thin films insoluble – an average conductivity of 5 × 10^−1^ S cm^−1^ is observed, which is unaffected by washing (ESI, Fig. S13†).

Analysis of the films of P3ZT by X-ray photoelectron spectroscopy (XPS) shows an increase in the work function from 4.0 eV to 4.9 eV after doping (ESI, Fig. S19†). The chemical composition of the surface was analysed: the Fe 2p and Cl 2p signals indicate successful doping. Washing removes iron(ii) and unreacted iron(iii) chloride as evidenced by the decrease of their signals in the XPS spectrum (ESI, Fig. S18†). Furthermore, chloride ions are detected as a counterion to the oxidized polythiophene backbone.

The doping process was transferred to the EBL-immobilized and – structured nanowires (Fig. 3a and b). To calculate the conductivity, the wires were described as half cylinders with a radius of 55 nm. The distance *W* between two gold contacts is 2.5 μm. A dependence on the applied dose is observed (1.5 to 3.5 mC cm^−2^). With increasing irradiation dose, the crosslinking degree increased, as does the conductivity initially (1.6 to 4.4 S cm^−1^) before reaching a plateau at a dose exceeding 3 mC cm^−2^. After washing, the conductivity was reduced by one order of magnitude to 0.5 (2.2 mC cm^−2^) or 0.7 S cm^−1^ (3.0 mC cm^−2^) for the patterned nanowires and by a factor of 20 to 0.15 S cm^−1^ for increased doses of 3.5 mC cm^−2^. This effect may be a consequence of the degradation induced by EBL as evidenced by the contrast change in the secondary electron images recorded in SEM (*vide supra*) and/or formation of a higher crosslinked network with increasing dose (ESI, Fig. S11†) impeding FeCl_3_ diffusion into the nanowires rendering the doping less effective. Stability and processing of doped organic nanowires is challenging as the dopant counterions are not covalently bound to the polythiophene, but diffuse freely in an applied electric field and drift.^40^ Likewise, unreacted dopant (from redox equilibria) can be removed from the film by polar solvents. Impedance measurements quantified dopant drift. An ohmic resistor or ideal conductor shows a frequency independent capacitance. A change in *C*′ corresponds to a drift of the dopants. Characteristically, this occurs mainly in the unwashed iron(iii) chloride doped, EBL structured samples. In crosslinked P3ZT/FeCl_3_ samples drift was absent (Fig. S14†).

These lithographically fabricated wires are promising electrodes for fully organic nanoscale field-effect transistors (nFETs). Electrodes for all organic transistor have so far been developed based on carbon nanotubes, PEDOT/PSS, or doped polypyrrole.^41–49^ Only a small number of these transistors have achieved resolutions smaller than a few microns.^42^ Thus, different sizes of interdigitating finger electrodes were written at a dose of 2.5 mC cm^−2^ (for the fingers) and 1.5 mC cm^−2^ (for the contact pads). The line width of the finger contacts was varied from 20, 50, 100 to 200 nm. Ideally, the channel width is 1.8 μm and the length is 500 nm. However, the proximity effect limited the resolution to 100 nm, reducing the effective channel length to (410 ± 5) nm. The real dimensions of the transistor were determined by SEM (see ESI, Fig. S12† and Table S1†). The electrodes were fabricated on doped silicon wafers with a 100 nm SiO_2_ insulating top layer functionalized with a self-assembled monolayer of 12-cyclohexyldodecylphosphonic acid (CDPA). The self-assembled monolayer should not influence processing parameters for electron beam lithography, but the SAM influences the film-forming properties of TIPS-Pen and therefore increases the performance of our nFET (Table 2).

**Table 2:** Averaged over a number of six devices and top parameters and metrics of nanostructured field-effect transistors and reference Au-based transistors

TIPS-Pen	Mobility [cm^2^ V^−1^ s^−1^]	On/off voltage [V]	On/off ratio *I*_D_
nFET_avg._	(1.7 ± 1.4) × 10^−4^	−2.0 ± 1.8	680 ± 220
nFET_top_	4.7 × 10^−4^	−0.5	1100
ref_avg._	(4.5 ± 3.7) × 10^−4^	−0.2 ± 0.15	730 ± 205
ref_top_	8.0 × 10^−4^	−0.35	1020

**Fig. 4:**
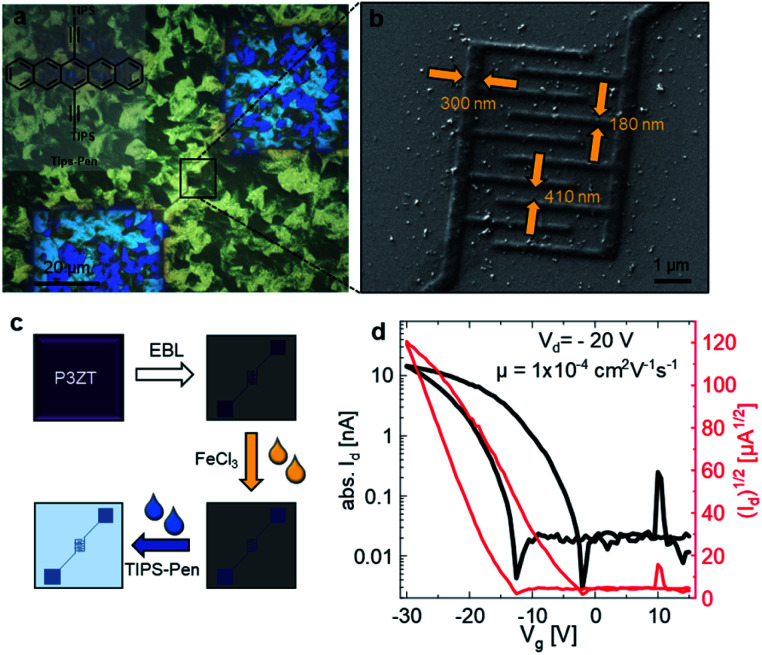
(a) Polarizing microscope image of EBL nanostructured P3ZT on a SiO_2_ substrate forming a transistor structure. Covered by a film of TIPS-Pen (inset). (b) SEM images of the nanotransistor. (c) Processing steps towards the nanotransistor and (d) transfer characteristics of the nanosized transistors.

Fig. 4a shows micrographs with cross-polarization of the bottom-gate bottom-contact nanotransistor, fabricated by spincoating TIPS-Pen from toluene (10 mg L^−1^) on top of the conductive polymer nanostructures (for an illustration of the fabrication process, see Fig. 4c). TIPS-Pen was chosen due to its work function (4.7 eV)^50^ in close proximity to that of doped and immobilized P3ZT. A SEM image of the interdigitated contacts of a transistor on silicon oxide is shown in Fig. 4b revealing the dimensions of the interdigitated contacts of the nFET. Fig. 4d shows an exemplary transfer characteristic of the nanostructured transistors. Despite the significantly reduced transistor dimensions in comparison to conventional organic field-effect transistors, the device shows typical behaviour of a p-type OFET, albeit with some hysteresis and a slightly smaller ratio between on/off currents *I*_D_. A maximum mobility of 4 × 10^−4^ cm^2^ V^−1^ s^−1^ was calculated, on par to our reference transistor. The hysteresis can be attributed to deep traps at the interface of SiO_2_/organic semiconductor. The output characteristics indicates the presence of injection barriers and trap states, too (ESI, Fig. S17b†). Compared to a reference transistor with gold electrodes, higher drain voltages *V*_D_ are necessary due to the poorer conductivity of the contacts (ESI, Fig S17a†). For comparison, mobilities in the order of 10^−4^ cm^2^ V^−1^ s^−1^ were calculated for spin-coated TIPS-Pen on pre-structured transistors (Au, *W* = 10 mm, *L* = 5 μm, for TFT characteristics see Fig. S15†) at lower drain voltages (*V*_d_ = −10 V).

Based on the measured dimensions of the fabricated electrode, the resistances of the electrode in the transistor was estimated using the previously determined conductivities for FeCl_3_-doped P3ZT. The calculated resistance of the finger structures still contributes <1% to the total resistance of the transistor. Thus, the small difference in OFET mobility between the gold and P3ZT/FeCl_3_ is not caused by the electrode's bulk properties but rather by interface effects between the semiconductor and the electrode.

## Conclusions

In summary, we reduced an electronic device, namely a transistor, to a total size of a few micrometers using a novel polythiophene with crosslinkable cinnamate side-chains. Electron irradiation desolubilizes thin films of P3ZT – lithography enables patterning of nanowires, conducting after doping. Electron beam lithography induced [2+2] cycloaddition of cinnamate side-chains allows the free design of all-organic circuits. Further miniaturization is within the possibilities of the concept presented herein.

## Data availability

Data related to this article are available through heiDATA, the institutional research data repository of Heidelberg University, under https://heidata.uni-heidelberg.de/dataverse/oci-bunz-group.

## Author contributions

All experiments were performed by N. M. B. and C. H. K.-P. S. and N. N. B. analysed conductivity and transistor performance. R. B: provided XPS and UPS-measurements. L. V. and I. W. performed electron microscopy of the cross sections. N. M. B. and J. F. drafted the first version of the paper. C. M., R. R. S, P. T. and U. H. F. supervised this project. All the authors contributed to the interpretation of the results and writing of the manuscript.

## Conflicts of interest

There are no conflicts to declare.

## Supplementary Material

SC-013-D2SC01867E-s001
