# Supplementary material for: Electron-beam lithography of cinnamate polythiophene films: conductive nanorods for electronic applications
Source: Chem Sci. 2022 Jun 16;13(26):7880–5. doi: 10.1039/d2sc01867e (PMC9258344; doi:10.1039/d2sc01867e)

Electronic Supplementary Material (ESI) for Chemical Science.
This journal is © The Royal Society of Chemistry 2022

Supporting Information

Electron-beam lithography of cinnamate polythiophene films: Conductive nanorods for electronic applications

N. Maximilian Bojanowski,[a] Christian Huck,[b,c] Lisa Veith,[b] Karl-Philipp Strunk,[d] Rainer Bäuerle,[a,d] Christian Melzer,[d] Jan Freudenberg,[a] Irene Wacker,[b] Rasmus R. Schröder,[b] Petra Tegeder,[b,c] Uwe H. F. Bunz [a,b]*

a. Organisch-Chemisches Institut, Ruprecht-Karls-Universität Heidelberg, Im Neuenheimer Feld 270, 69120 Heidelberg,
b. Centre for Advanced Materials, Ruprecht-Karls-Universität Heidelberg, Im Neuenheimer Feld 225, 69120 Heidelberg.
c. Physikalisch-Chemisches Institut, Ruprecht-Karls-Universität Heidelberg, Im Neuenheimer Feld 253, 69120 Heidelberg
d. InnovationLab GmbH, Speyerer Straße 4, 69115 Heidelberg, Germany,
E-mail: uwe.bunz@oci.uni-heidelberg.de

Table of Content

1. General information.....2
2. Synthetic and experimental procedures.....4
3. Additional spectra.....8
4. Film retention.....10
5. Additional scanning electron micrographs.....11
6. Conductivity measurements and transistor characteristics.....14
7. XPS and UPS spectra.....17
8. References.....18
9. NMR spectra.....19

1. General information

For experiments conducted under an atmosphere of nitrogen, standard Schlenk and glovebox (Unilab-2000, MBRAUN) techniques were used. The addition of solvents or reagents was carried out using nitrogen flushed stainless steel cannulas and plastic syringes. THF was dried over sodium and freshly distilled before use. P3HT (LOT = M107, M_w = 2.4 x 10^4 g/mol) was purchased from Ossila Ltd. For spectroscopic and analytic characterizations, the following devices were used:

^{1}H NMR spectra were recorded at room temperature on the following spectrometers: Bruker AC-300 (300.51 MHz), Bruker Avance 500 (500.13 MHz) or Bruker Avance 600 (600.25 MHz). Chemical shifts (δ) were measured relatively to the shift of residual protons in deuterated solvents or relatively to an internal standard and values are given in parts per million (ppm). Coupling constants (J) are stated in Hertz (Hz). Signal patterns are noted according to usual conventions (s=singlet, d=doublet, t=triplet, q=quartet, quin=quintet, sext=sextet, dd=doublet of doublet, dt=doublet of triplet, ddd=doublet of doublet of doublet, etc., br. s=broad signal, m=multiplet).

^{13}C NMR spectra were recorded at room temperature on the following spectrometers: Bruker Avance 300 (75.48 MHz), Bruker Avance 500 (125.76 MHz) or Bruker Avance III (150.93 MHz). Chemical shifts (δ) were measured relatively to the solvent signals or relatively to an internal standard and values are given in parts per million (ppm). ^{13}C NMR spectra were collected using proton decoupling algorithms.

High resolution mass spectra (HR-MS) were either recorded on a Bruker ApexQehybrid 9.4 T FT-ICR-MS (ESI+, DART+), a Finnigan LCQ (ESI+) or a JEOL JMS-700 (EI+) mass spectrometer at the Institute of Organic Chemistry of Heidelberg University.

Elemental analysis was performed by the Microanalytical Laboratory of the University of Heidelberg using an Elementar Vario Micro Cube.

Analytical gel permeation chromatography (GPC) was performed using a JASCO PU 2080 HPLC pump equipped with three different commercially available **P**olymer **S**tandards **S**ervice (PSS) cross-linked poly(stryrene) columns (5 µm particle size, L × I.D. 30 cm × 8 mm) in line. The consecutive exclusion sizes were 100 Å, 1000 Å, and 100000 Å respectively. For detection a JASCO UV-2075 Plus detector and a JASCO RI-2031 detector were used. The column was operated with chloroform as eluent and a flow rate of 1.0 mL/s.

UV/VIS spectra were recorded on a Jasco UV-VIS V-660 or Jasco UV-VIS V-670 and processed with the software JASCO Spectra Manager™ II.

Scanning electron microscopy (SEM) images: The surface of the sample (silicon wafer with gold contacts) was electrically connected to the SEM carrier using sticky copper tape and silver paint. Samples were imaged without further coating to allow direct imaging of the materials and correlation of secondary electron yield (contrast variations) with materials properties such as work function. Images were recorded in a Crossbeam 540 (Carl Zeiss Microscopy, Oberkochen, Germany) at 1.5 keV landing energy using low probe current.

Atomic force microscopy (AFM) was carried out on a Bruker Nanoscope MultiMode VIII in the ScanAsyst PeakForce mode. The resulting images were treated with the software Gwyddion for row alignment and scar correction.

Conductivity measurements were carried out as 2-point measurements.

2. Synthetic and experimental procedures

Synthesis and characterization: 2-(2,5-Dibromthiophen-3-yl)ethan-1-ol (1.12 g, 3.92 mmol, 1 eq.) and triethylamine (TEA, 475 mg, 4.7 mmol, 1.2 eq.) were dissolved in dry DCM (10 mL) under nitrogen atmosphere and added to a flask containing cinnamon acid chloride (1.30 g, 7.83 mmol, 2 eq.). The mixture was stirred for 24 h, extracted with diethyl ether (2x 20 mL) and washed with water (50 mL). The solvent was removed in vacuo and the residue subjected to flash chromatography (SiO_2, petroleum ether/ethyl acetate 10:1). The solvent was removed in vacuo to give the title compound as a pale-yellow solid. The crude product was recrystallized in DCM to afford white crystals of **3** (1.35 g, 84%). Mp (dec.) > 250 °C. ^{1}H NMR (300 MHz, $CDCl_3$): δ = 7.72 (d, *J*=16.1 Hz, 1 H), 7.51 - 7.60 (m, 2 H), 7.33 - 7.47 (m, 3 H), 6.91 (s, 1 H), 6.45 (d, *J*=16.1 Hz, 1 H), 4.38 (t, *J*=6.7 Hz, 2 H), 2.98 (t, *J*=6.7 Hz, 2 H) ppm. ^{13}C{^{1}H} NMR (75 MHz, $CDCl_3$): δ = 166.7, 145.2, 138.4, 134.3, 131.1, 130.4, 128.9, 128.1, 117.8, 110.9, 63.0, 29.1 ppm. HR-MS (ESI+) m/z: $[M+Na]^+$ calcd. for $C_{15}H_{12}Br_2O_2SNa$ 438.8802; found 438.8797. Elem. anal. calcd. for $C_{15}H_{12}Br_2O_2S$: C 43.30 H 2.91; found C 43.19, H 3.14.

2-(2,5-Dibromothiophen-3-yl)ethyl cinnamate **3** (156 mg, 375 µmol, 1 eq.) was dissolved in dry THF (8 mL) and cooled to 0 °C under argon atmosphere. *i*PrMgCl (2 M in THF, 190 µL, 375 µmol, 1 eq.) was added dropwise and stirring was maintained for 1 h at 0 °C. The solution was warmed to room temperature and Ni(dppp)Cl_2 (1.00 mg, 1.87 µmol, 1 mol%) was added. The reaction was stirred under argon at 60 °C for 3 h. Saturated aqueous solutions of NH_4Cl and $CHCl_3$ were added, the aqueous layer was separated and extracted with $CHCl_3$ (3x 20 mL). The combined organic layers were dried over $MgSO_4$, filtered, and the filtrate concentrated *in vacuo*. The crude product purified by twofold precipitation via dissolution in a small amount of $CHCl_3$ and subsequent addition to methanol to afford the title compound as an orange solid.

After Soxhlet extraction (methanol, hexane, DCM and chloroform), the chloroform fraction was precipitated into methanol to afford **P3ZT** as a deep purple-golden solid (75.0 mg, 75%). M.p. 178 °C, dec. > 300 °C. The M_w was estimated to be 1.7 x 10^4 g/mol with a PDI of 1.74. ^{1}H NMR (500 MHz, $CDCl_3$): δ = ^{1}H NMR (300 MHz, $CDCl_3$) δ = 7.60 - 7.74 (m, 1 H), 7.48 (br s, 2 H), 7.35 (br s, 3 H), 7.14 (br s, 1 H), 6.32 - 6.49 (m, 1 H), 4.30 - 4.60 (m, 2 H), 3.13 - 3.27 (m, 1 H), 2.90 - 3.12 (m, 1 H) ppm. ^{13}C{^{1}H} NMR (100 MHz, $CDCl_3$): δ = 166.8, 145.1, 135.3, 134.3, 133.7, 132.4, 130.3, 129.3, 128.9, 128.1, 117.9, 63.9, 28.8 ppm.

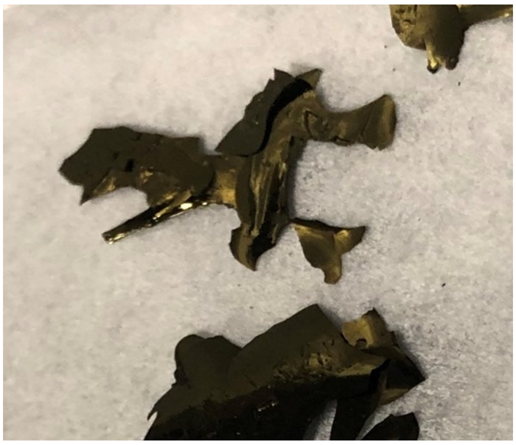

Figure S1. Picture of **P3ZT** under daylight.

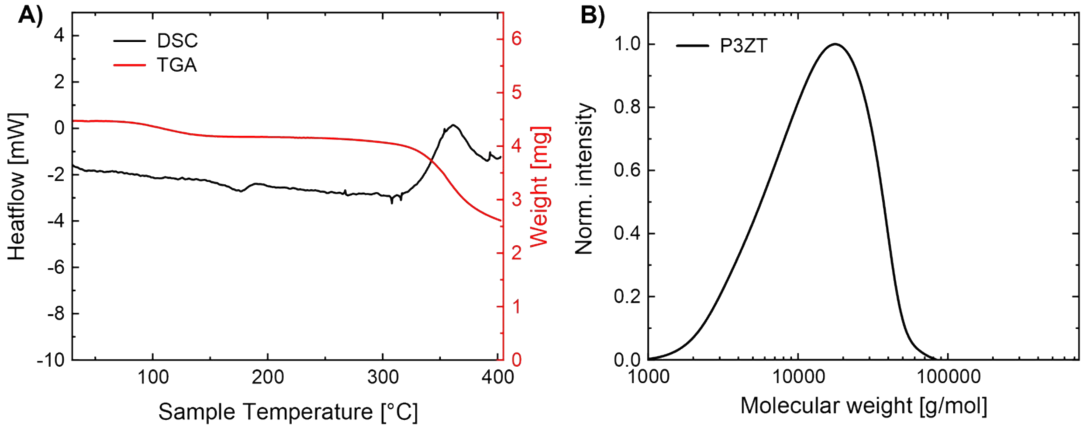

Figure S2. A) TGA and DSC and **B)** molecular weight distribution of **P3ZT**. Initial decrease in weight at 100 °C is attributed to solvent evaporation. DSC indicates a melting point at 178 °C.

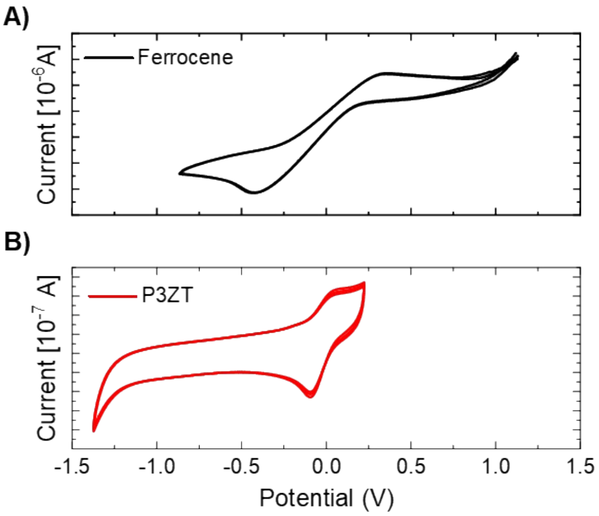

Figure S3. Cyclovoltammetry of **P3ZT** against an external standard (ferrocene) in THF.

Sample fabrication:

All wafers were successively cleaned via ultra-sonication in acetone, isopropanol, washed with water and dried in a stream of nitrogen:

a) undoped silicone wafers for IR transmission measurements
b) end-of-line substrates with 90 nm thermally grown SiO_2 and prefabricated Au contact pads
c) highly-doped silicon wafer with 100 nm thick thermally grown oxide for transistor applications

The undoped silicone wafer and the end-of-line substrate were cleaned via oxygen plasma prior to use. The highly doped silicon wafer was placed in Caro´s acid and was heated to 100 °C for 20 min. After cleaning with water and drying, a 150 mM solution of $Al(NO_3)_3 * 9\ H_2O$ in ethanol was spin-coated (5000 rpm; 40 s) onto the substrate. The wafer was directly heated to 300 °C for 30 min. For the formation of the self-assembled monolayer, the substrate was placed in a 15.0 mM solution of 12-cyclohexyldodecylphosphonic acid (CDPA) in isopropanol at room temperature for 16 h. Then the substrate was cleaned via ultra-sonication in isopropanol for 10 min, rinsed with water and dried in a stream of nitrogen. The capacitance of the dielectric layer amounted to 26 nF cm^{-2}.

P3ZT was spin coated on cleaned wafers from filtered solutions (10 mg/mL in chloroform) at 1000 rpm inside a nitrogen filled glovebox. Film thicknesses were measured using a Bruker DekTak XT profilometer.

UV crosslinking was done inside a nitrogen filled glovebox using a λ = 254 nm 15 W tube lamp from Herolab.

EBL: Spin-coated layers were nanopatterned using a Raith eLINE Plus nanofabrication system. Electrons with an energy of 15 keV and a beam current of 340 pA were used to structure the sample. Undoped silicon was used to enable the IR measurement. Squares (50x50 µm, used for IR transmission microscopy) as well as the USAF 1951 target (groups 10-15) were written on the sample with exposure doses ranging from 400 to 5000 $\mu C/cm^2$. Contact pads of the transistor structure were written with an exposure dose of 1500 $\mu C/cm^2$ and finger structures were additionally stitched to contact pads with an exposure dose of 2500 $\mu C/cm^2$. After exposure, unexposed material was removed by development in chloroform under ambient conditions.

IR transmission microscopy of films required a transparent (undoped) silicon wafer as a substrate. Large-area samples irradiated with UV light (254 nm) were measured using a FTIR spectrometer (Bruker IFS 66v/s). Samples were illuminated by unpolarized light emitted from a thermal light source. Transmitted light was collected with a liquid nitrogen cooled mercury cadmium telluride (MCT) detector. The whole beam path was evacuated to avoid atmospheric water and carbon dioxide absorption. Spectra were recorded with a resolution of 4 cm^{-1} and 200 scans. The transmittance of the layer was normalized to that of the bare substrate to get the relative transmittance spectra. Small-area samples produced by EBL were characterized using a Bruker Tensor 27 spectrometer coupled to a Hyperion 1000 IR microscope. Light was focused onto the sample and collected using two 36x reverse Cassegrain objectives. The whole beam path was purged with dried air to avoid atmospheric water and carbon dioxide absorption. Exposed layers were located using white light microscopy and centered in the IR beam whose

extent was specified by a circular aperture with a diameter of 50 µm. Spectra were recorded with a resolution of 2 cm^{-1} and at least 2000 scans.

Doping process and transistor fabrication:

The resulting nanorods were sequentially doped via spin-coating of a $FeCl_3$ solution in DMF (1 mg/mL) according to literature.[1] The doped structures were washed with acetonitrile after doping. **TIPS-Pen** was spin-coated from 10 mg/mL in toluene at 1000 rpm.

Devices were measured inside a nitrogen-filled glovebox using a commercially probe card station with a Keithly 4200-SCS parameter analyzer.

3. Additional spectra

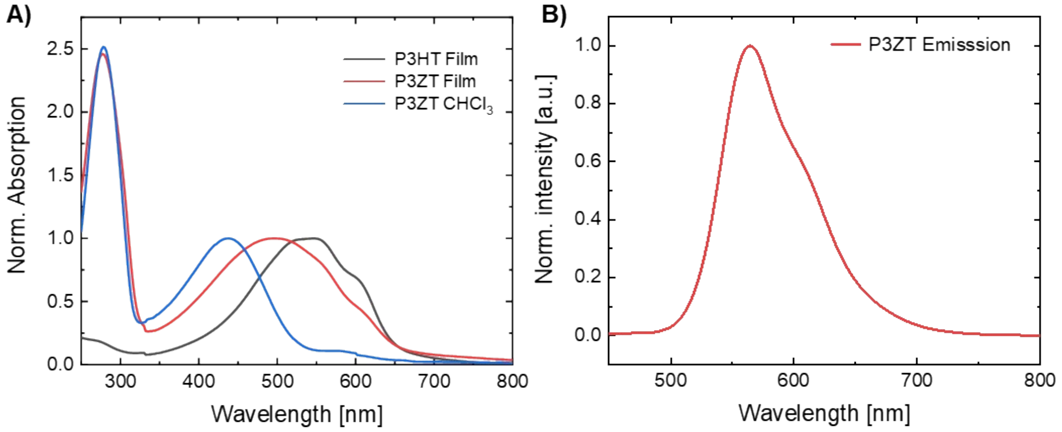

Figure S4. A) Normalized absorption spectra of **P3ZT** film and solution and **P3HT** film and **B)** emission spectra of **P3ZT** in solution ($CHCl_3$).

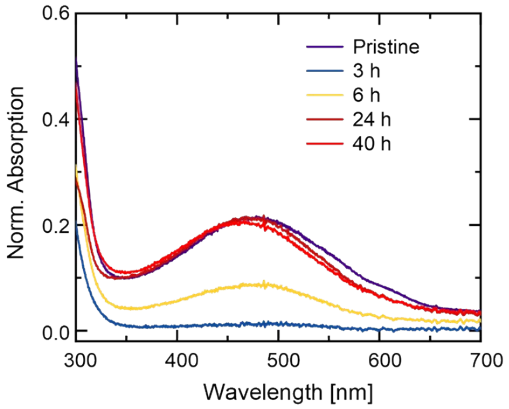

Figure S5. Absorption spectra after UV irradiation for prolonged times at 254 nm.

Complete set of IR spectra:

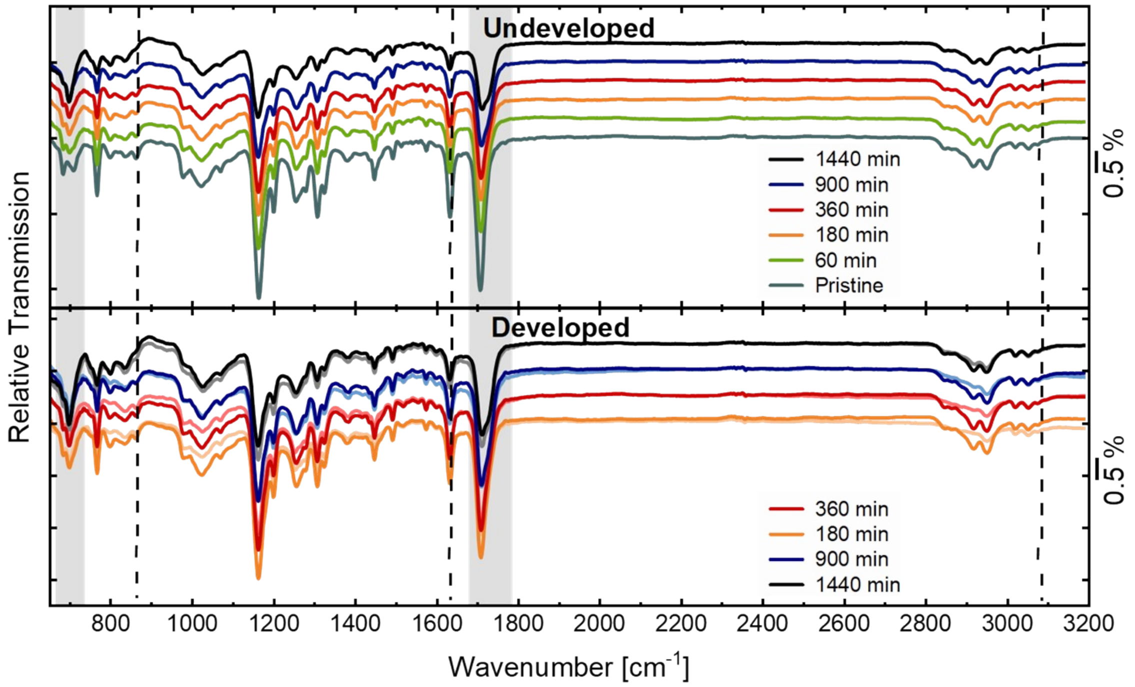

Figure S6. Complete set of IR spectra for cross-linking of **P3ZT** through UV light radiation. Comparing the transmission spectra before and after development as indicated by the dotted lines, reveals minimal loss of material further proving a complete desolubilization.

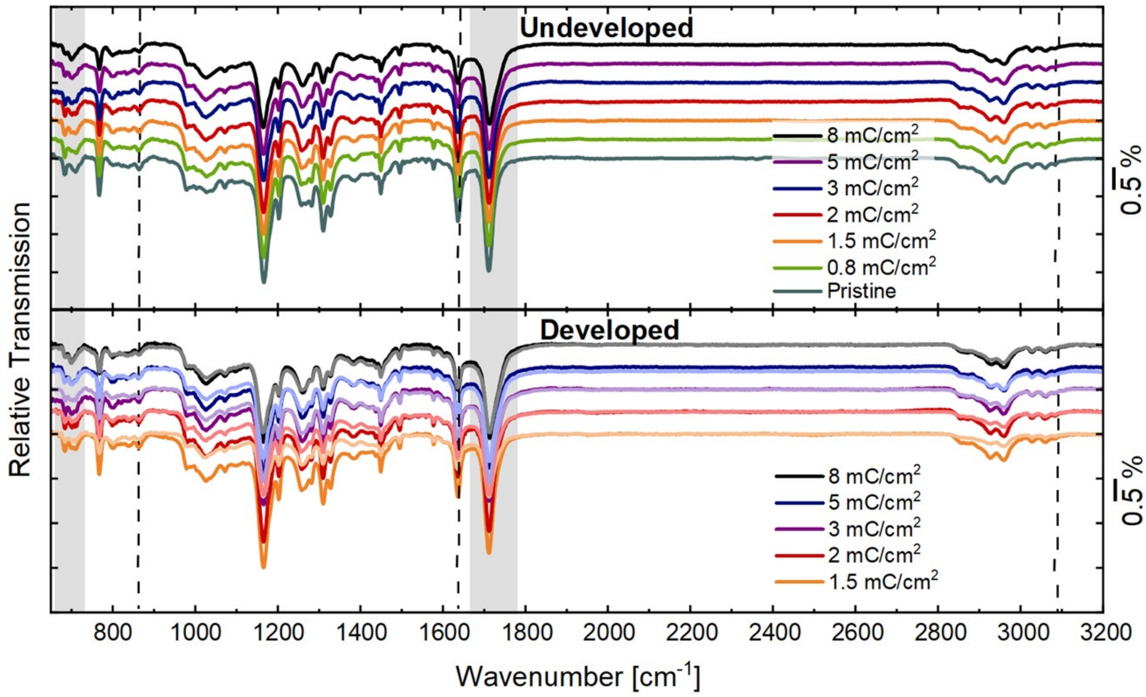

Figure S7. Complete set of IR spectra for cross-linking of **P3ZT** through electron beam radiation.

.

4. Film retention

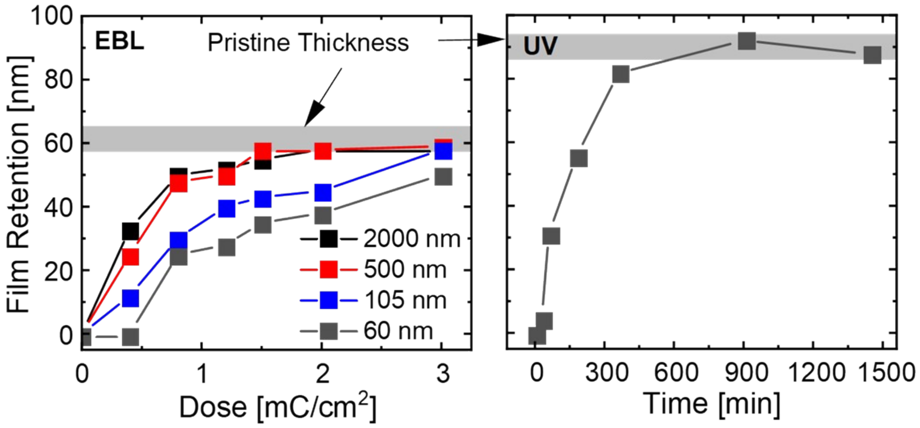

Figure S8. Film retention curve (film thickness after development) for cross-linking of **P3ZT** through EBL irradiation (left) and UV radiation (right). EBL was done at 10 keV where the thickness was measured at structures of different horizontal extent (smallest dimension given).

5. Additional scanning electron micrographs

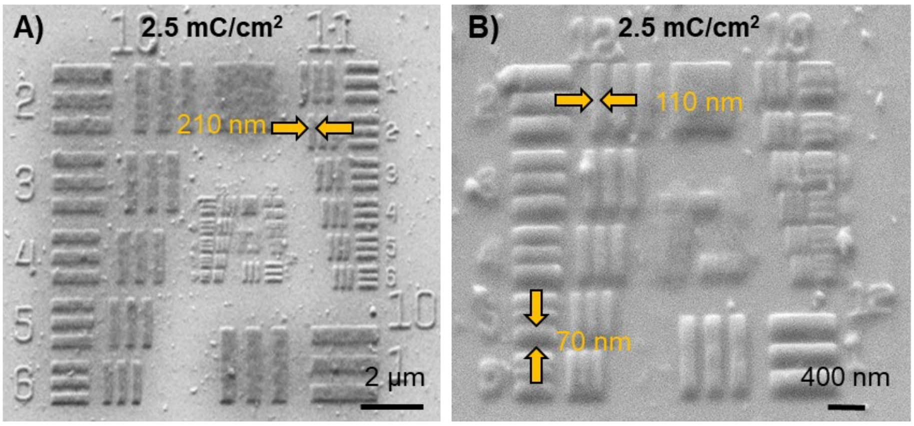

Figure S9. A) SEM images of a structure written at 2.5 mC/cm^2. **B)** The 12-5 group in the bottom left of the SEM still exhibits trenches between the bars of nominal width of 68 nm. Starting with group 13-1 (top right, nominal width 61 nm) the trenches start to disappear.

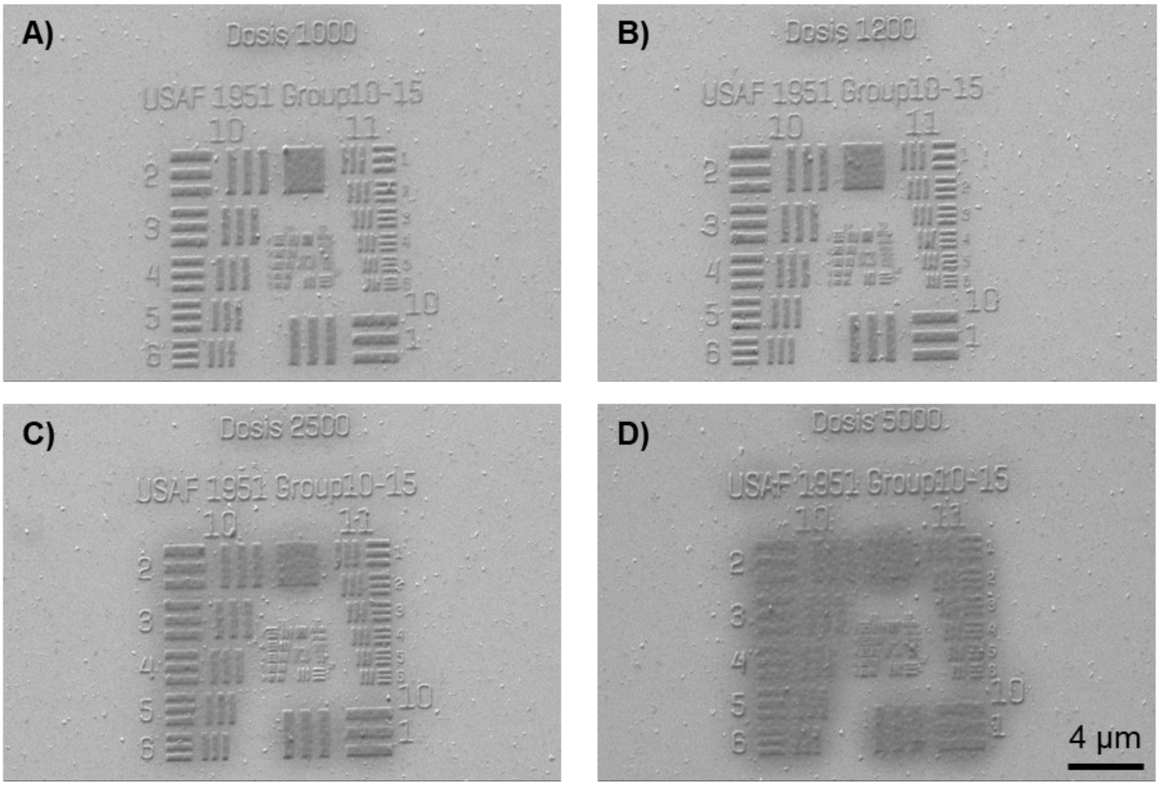

Figure S10. SEM images (SESI Detector) of structures of **P3ZT** written at **A)** 1.0 mC/cm^2, **B)** 1.2 mC/cm^2, **C)** 2.5 mC/cm^2, and **D)** 5.0 mC/cm^2. An over exposure is apparent at highest dose.

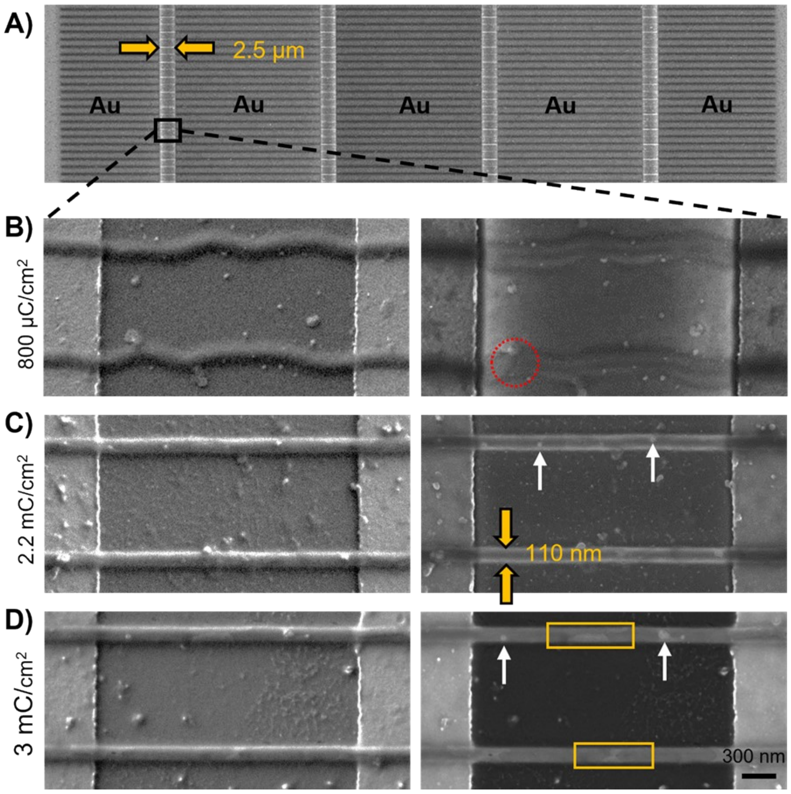

Figure S11. EBL nanostructured **P3ZT** on pre-patterned end-of-line substrates as seen in secondary electron SEM images. **A)** Overview image of parallel lines across multiple gold contacts. **B-D)** Magnified section of individual lines written by different EBL electron dose (0.8 to 3 mC/cm^2). Left side: SESI detector, right side: In-lens detector. While the SESI detector is largely sensitive to topology (note the 3D effect in the images) the In-lens detector contrast reflects the yield of secondary electrons, which can be attributed to the work function. In **B)** interruptions of the lines are visible (red circle). In **D)** variations of contrast along the lines are visible (orange rectangles), which are different from the variations due to contamination (white arrows)

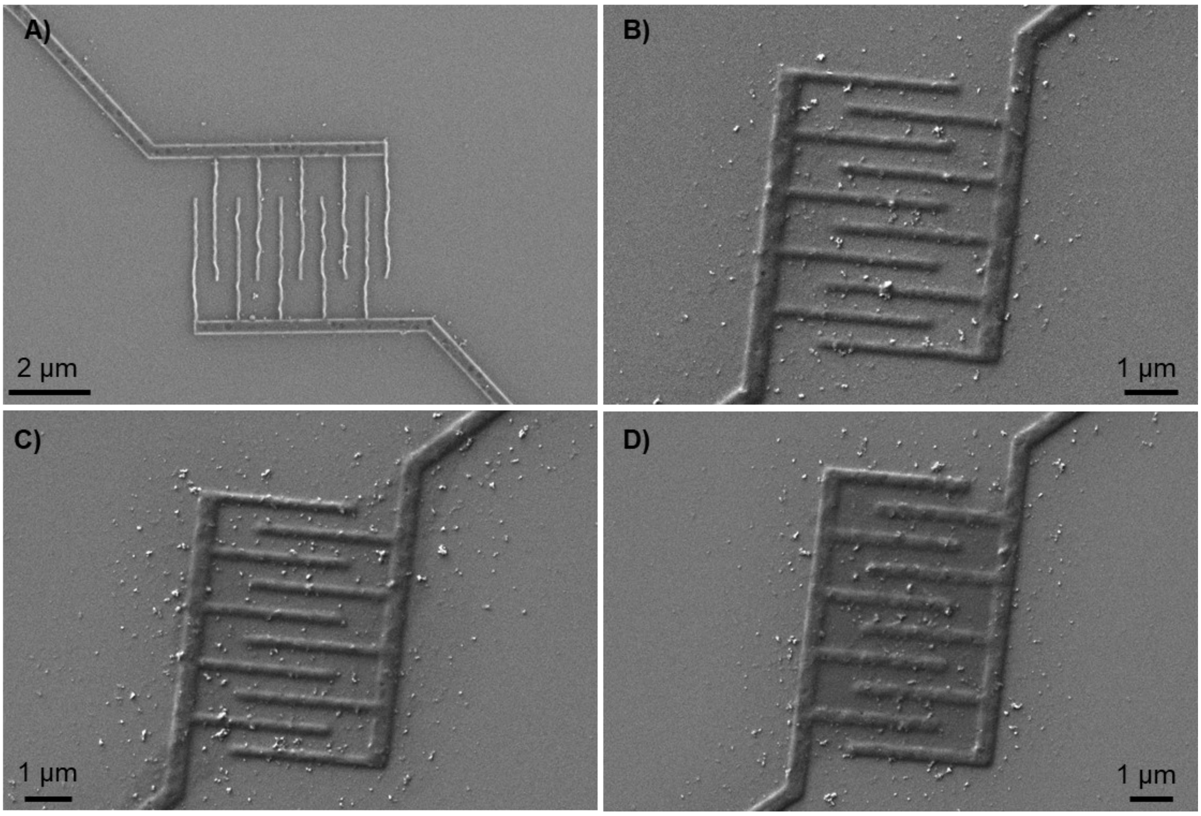

Figure S12. EBL nanostructured **P3ZT** towards a nanotransistor for different sizes of finger structure: A = 20 nm, B = 50 nm, C = 100 nm and D = 200 nm. The channel length L (500 nm) and width W (2.5 µm) are designed to be constant (see Table S1).

Table S1. Theoretical dimensions of the nanotransistor ($L_{theor.}$ = 500 nm) and those determined by SEM imaging.

Contact size (theo.) [nm]	Contact size (meas.) [nm]	Channel length (meas.) [nm]
20*	70 ± 5	400 ± 10
50	210 ± 10	350 ± 10
**100**	**180 ± 20**	**410 ± 5**
200§	220 ± 10	450

* Lines are inhomogeneous. § Overexposed structures.

6. Conductivity measurements and transistor characteristics

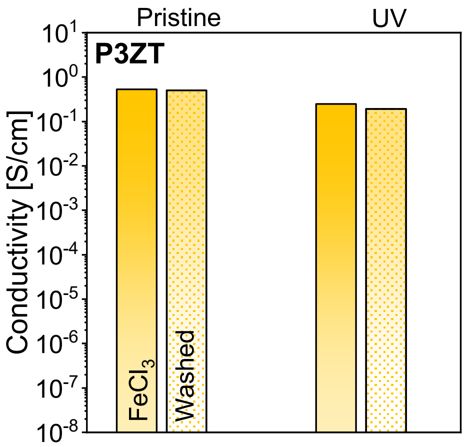

Figure S13. Conductivity of pristine and UV-crosslinked films of **P3ZT**. After doping with $FeCl_3$ the sample was washed.

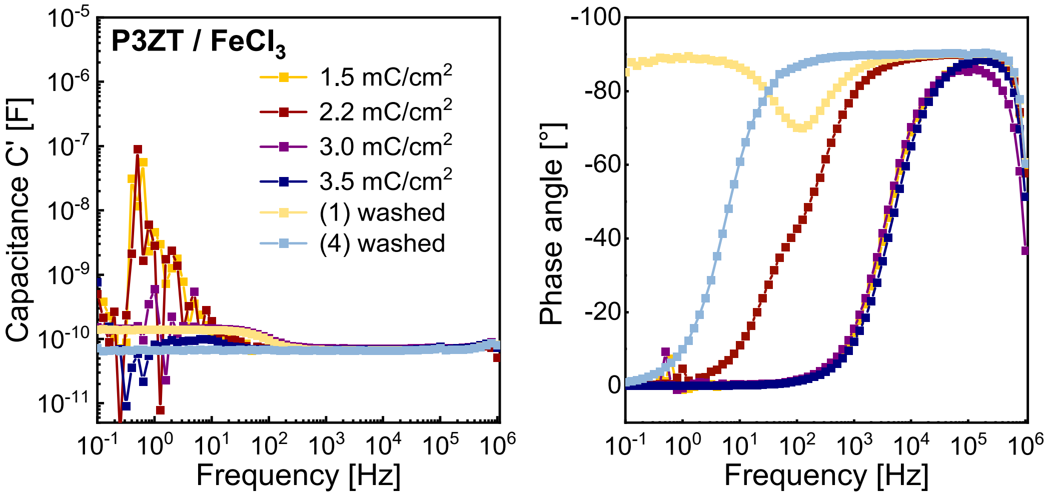

Figure S14. Impedance measurements of the conductive nanowires after doping with $FeCl_3$. Shown is the complex value of the capacitance and the phase shift as a function of frequency. In lighter colors the corresponding washed samples are shown.

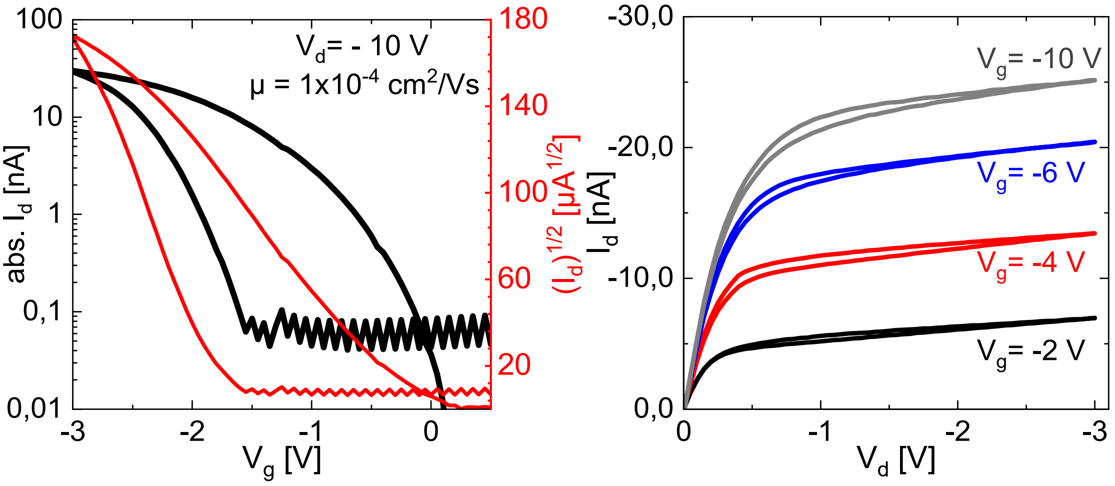

Figure S15. Input and output characteristics of TIPS-Pen.

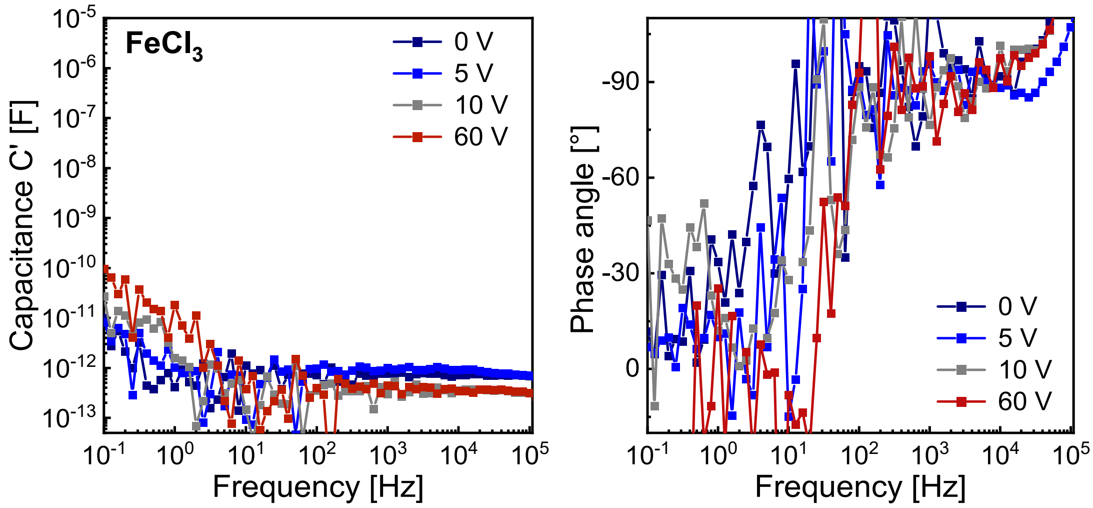

Figure S16. Impedance measurements of the nanotransistor after doping with $FeCl_3$ for different applied DC voltages V_D.

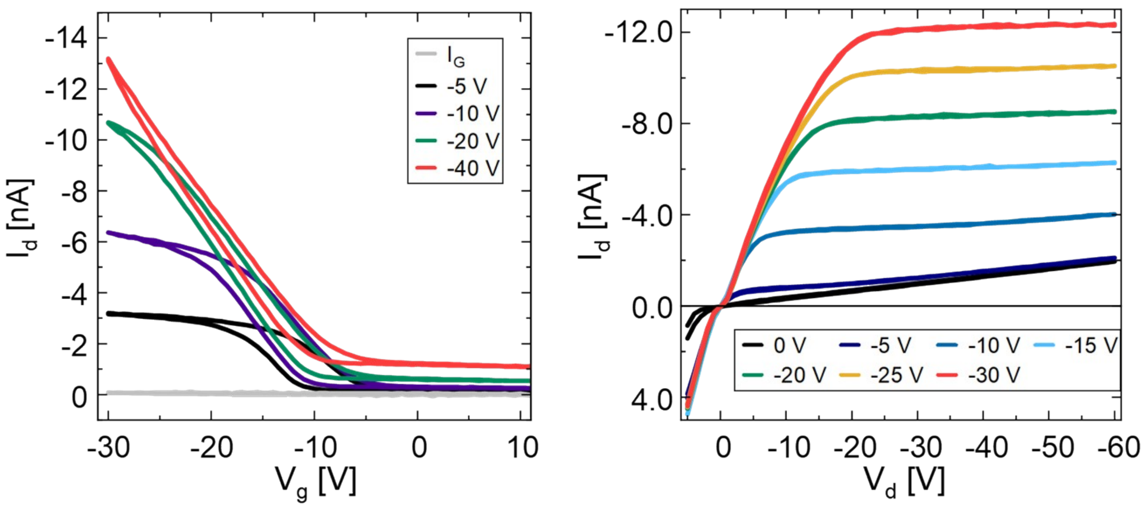

Figure S17. Input characteristic of TIPS-Pen of the nanotransistor for different drain voltages and output characteristic of the same nanotransistor for different gate voltages.

7. XPS and UPS spectra

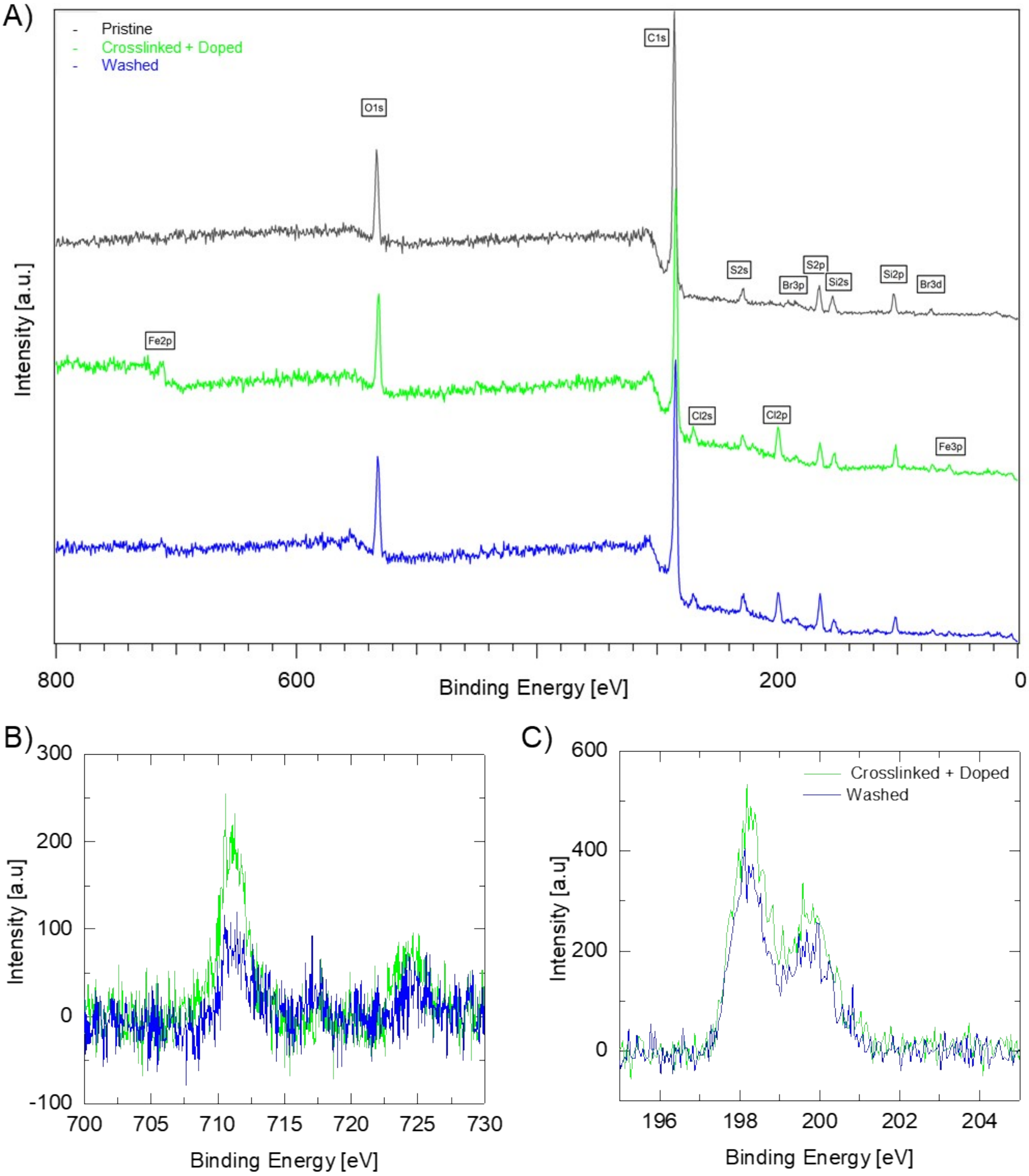

Figure S18. XPS spectra of **P3ZT** on Si/SiO_2 substrate. **A)** Survey of the whole spectra for a pristine sample (black), for a UV crosslinked (3h) and $FeCl_3$-doped sample (green) and washed as described with MeCN (blue). **B)** The Fe2p and **C)** Cl2p signals detailed for crosslinked/doped and for washed samples.

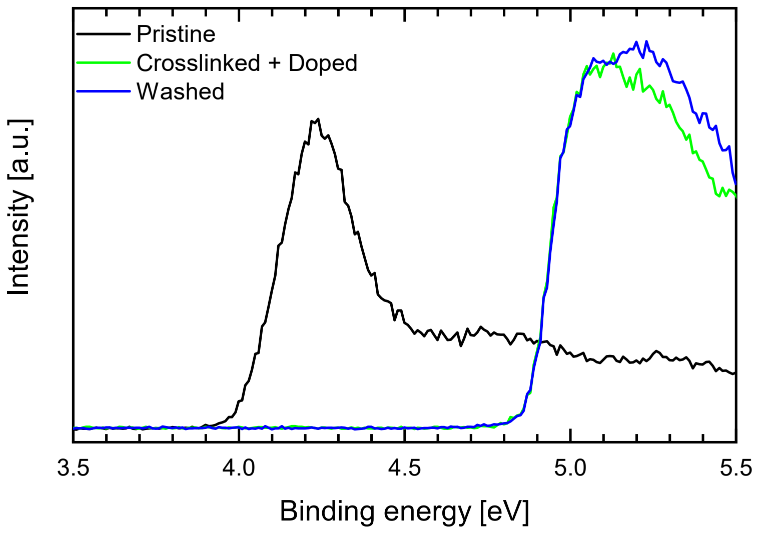

Figure S19. Secondary electron cut-off (SEC) from XPS to determine the work function (WF) of pristine **P3ZT**, of a UV crosslinked (3h) and $FeCl_3$-doped sample (green) and washed with MeCN (blue). The intersection of the tangent with the x-axis is determined.

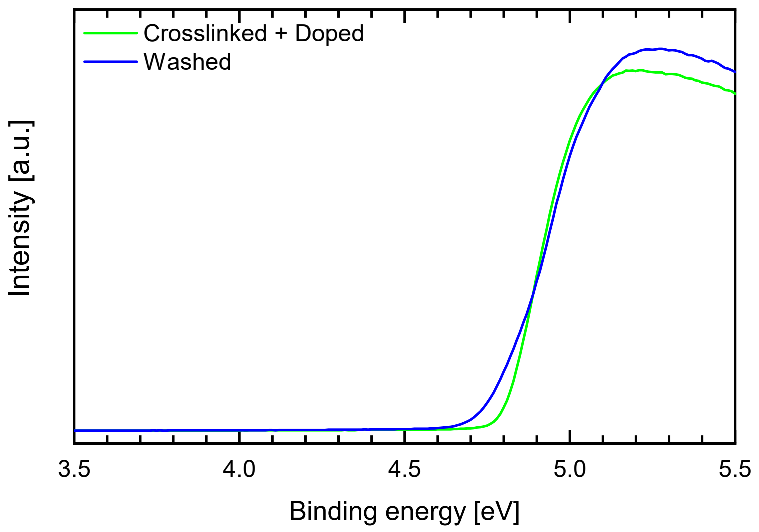

Figure S20. Secondary electron cut-off (SEC) from UPS to determine the WF of a UV crosslinked (3h) and $FeCl_3$-doped sample (green) and washed with MeCN (blue). The intersection of the tangent with the x-axis is determined.

8. References

1 I. E. Jacobs, E. W. Aasen, J. L. Oliveira, T. N. Fonseca, J. D. Roehling, J. Li, G. Zhang, M. P. Augustine, M. Mascal and A. J. Moulé, *J. Mater. Chem. C*, 2016, **4**, 3454–3466.

9. NMR spectra

1H NMR spectra of 3

Acquisition Time (sec)	4.0265	*Comment*	NB 529	*D*	0.1	*D1*	0.1	*DE*	6.5
DS	2	*Date*	16 Jan 2020 04:59:10			*Date Stamp*	16 Jan 2020 04:59:10		
File Name	\\bunz22\Mitarbeiter\Bojanowski\Doktorarbeit\Analytics\NMR\f200115ubnb.529\2\PDATA\1\1r					*Frequency (MHz)*	300.1600	*GB*	0
INSTRUM	<FOURIER300>			*LB*	0.3	*NS*	128	*Nucleus*	1H
Number of Transients	128	*Origin*	FOURIER300			*Original Points Count*	24576	*Owner*	ns
PC	1	*PROBHD*	<5 mm DUL 13C-1H/D Z-GRD Z111650/0010 >			*PULPROG*	<zg30>	*Points Count*	65536
Pulse Sequence	zg30	*Receiver Gain*	160.34	*SF*	300.16	*SFO1*	300.1615008		
SI	65536	*SSB*	0	*SW(cyclical) (Hz)*	6103.52	*SWH*	6103.515625		
Solvent	CHLOROFORM-d			*Spectrum Offset (Hz)*	1524.0314	*Spectrum Type*	standard	*Sweep Width (Hz)*	6103.42
TD	49152	*TD0*	1	*TE*	299.9	*Temperature (degree C)*	26.900	*UNC1*	<1H>
WDW	1								

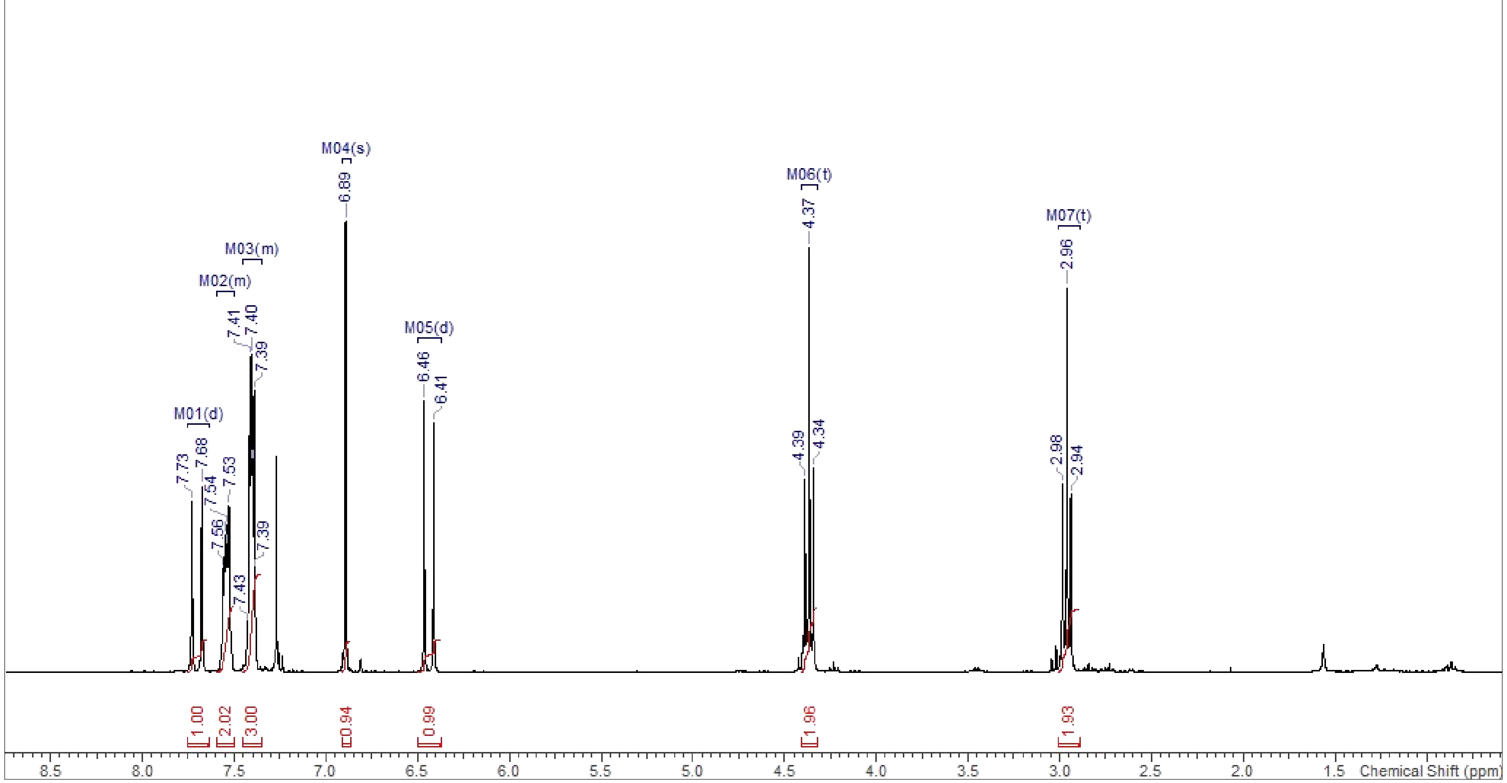

^{13}C NMR spectra of 3

Acquisition Time (sec)	1.3422	***Comment***	NB 529	***D***	0.03	***D1***	2	***DE***	6.5
DS	2	***Date***	15 Jan 2020 19:21:12			***Date Stamp***	15 Jan 2020 19:21:12		
File Name	\\bunz22\Mitarbeiter\Bojanowski\Doktorarbeit\Analytics\NMR\f200115ubnb.529\1\PDATA\1\1r					***Frequency (MHz)***	75.4753	***GB***	0
INSTRUM	<FOURIER300>			***LB***	1	***NS***	10240	***Nucleus***	13C
Number of Transients	10240	***Origin***	FOURIER300			***Original Points Count***	32768	***Owner***	ns
PC	1.4	***PROBHD***	<5 mm DUL 13C-1H/D Z-GRD Z111650/0010 >			***PULPROG***	<zgpg30>	***Points Count***	32768
Pulse Sequence	zgpg30	***Receiver Gain***	501.19	***SF***	75.475292032			***SFO1***	75.4835943141236
SI	32768	***SSB***	0	***SW(cyclical) (Hz)***	24414.06	***SWH***	24414.0625	***Solvent***	CHLOROFORM-d
Spectrum Offset (Hz)	8302.2842	***Spectrum Type***	standard	***Sweep Width (Hz)***	24413.32	***TD***	65536	***TD0***	1
TE	300.1	***Temperature (degree C)***	27.100	***UNC1***	<13C>	***WDW***	1		

f200115ubnb.529.001.001.1r

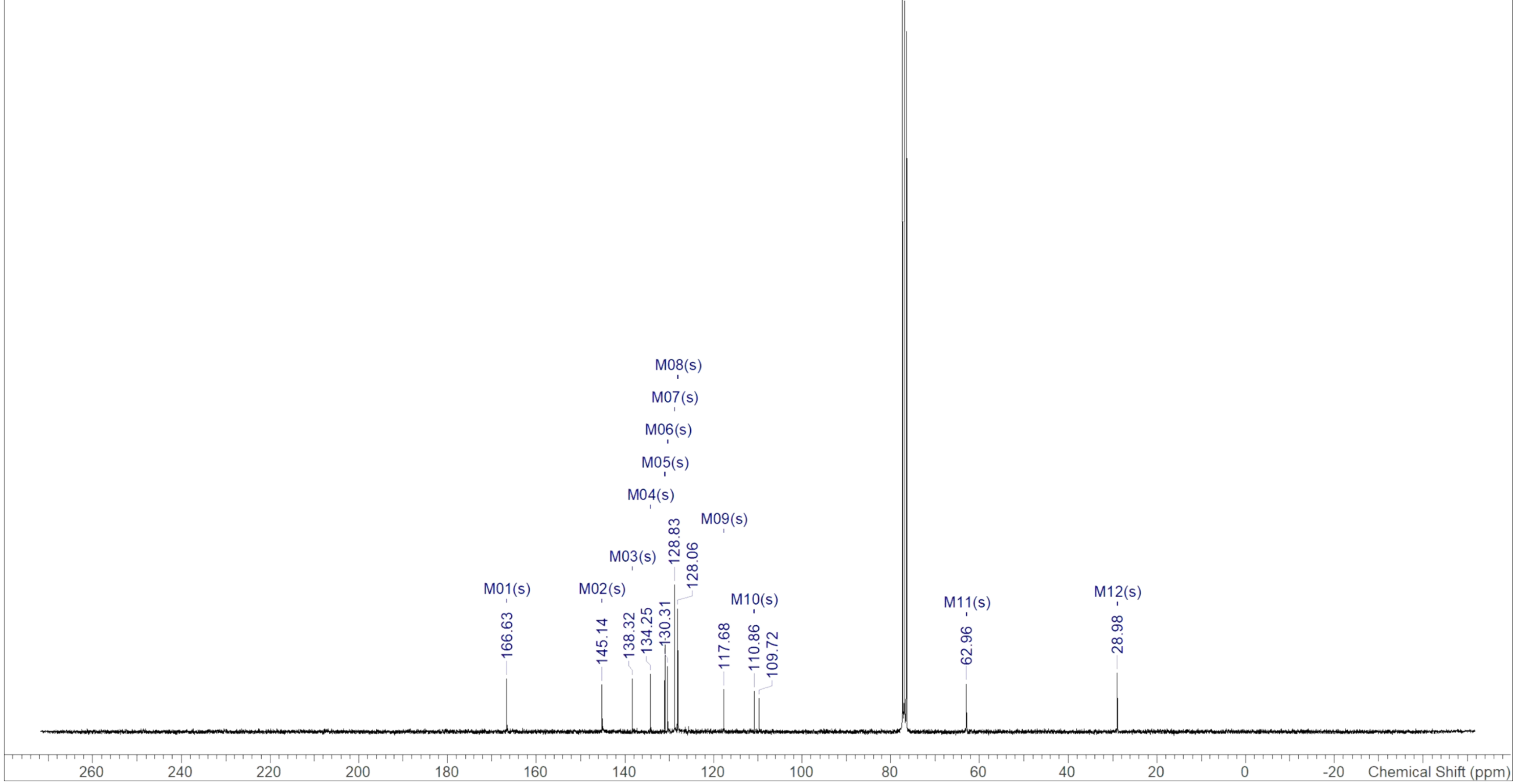

^{1}H NMR spectra of **P3ZT**

Acquisition Time (sec)	3.6351	*Comment*	Z104275 0201 (PA BBO 300S1 BBF-H-D-05 Z)			*D*	0.1	*D1*	0.1
DE	6.5	*DS*	2	*Date*	23 Jan 2020 16:12:01			*Date Stamp*	23 Jan 2020 16:12:01
File Name	\\bunz22\Mitarbeiter\Bojanowski\Doktorarbeit\Analytics\NMR\a200123ubnb234\1\PDATA\1\1r					*Frequency (MHz)*	300.5100	*GB*	0
INSTRUM	<spect>	*LB*	0.3	*NS*	16	*Nucleus*	1H	*Number of Transients*	16
Origin	spect	*Original Points Count*	32768	*Owner*	ns	*PC*	1		
PROBHD	<Z104275 0201 (PA BBO 300S1 BBF-H-D-05 Z)>			*PULPROG*	<zg30>	*Points Count*	65536	*Pulse Sequence*	zg30
Receiver Gain	406.00	*SF*	300.510030343375			*SFO1*	300.51150255		
SI	65536	*SSB*	0	*SW(cyclical) (Hz)*	9014.42	*SWH*	9014.42307692308		
Solvent	CHLOROFORM-d			*Spectrum Offset (Hz)*	1498.4125	*Spectrum Type*	standard	*Sweep Width (Hz)*	9014.29
TD	65536	*TD0*	2	*TE*	300.0021	*Temperature (degree C)*	27.002	*UNC1*	<1H>
WDW	1								

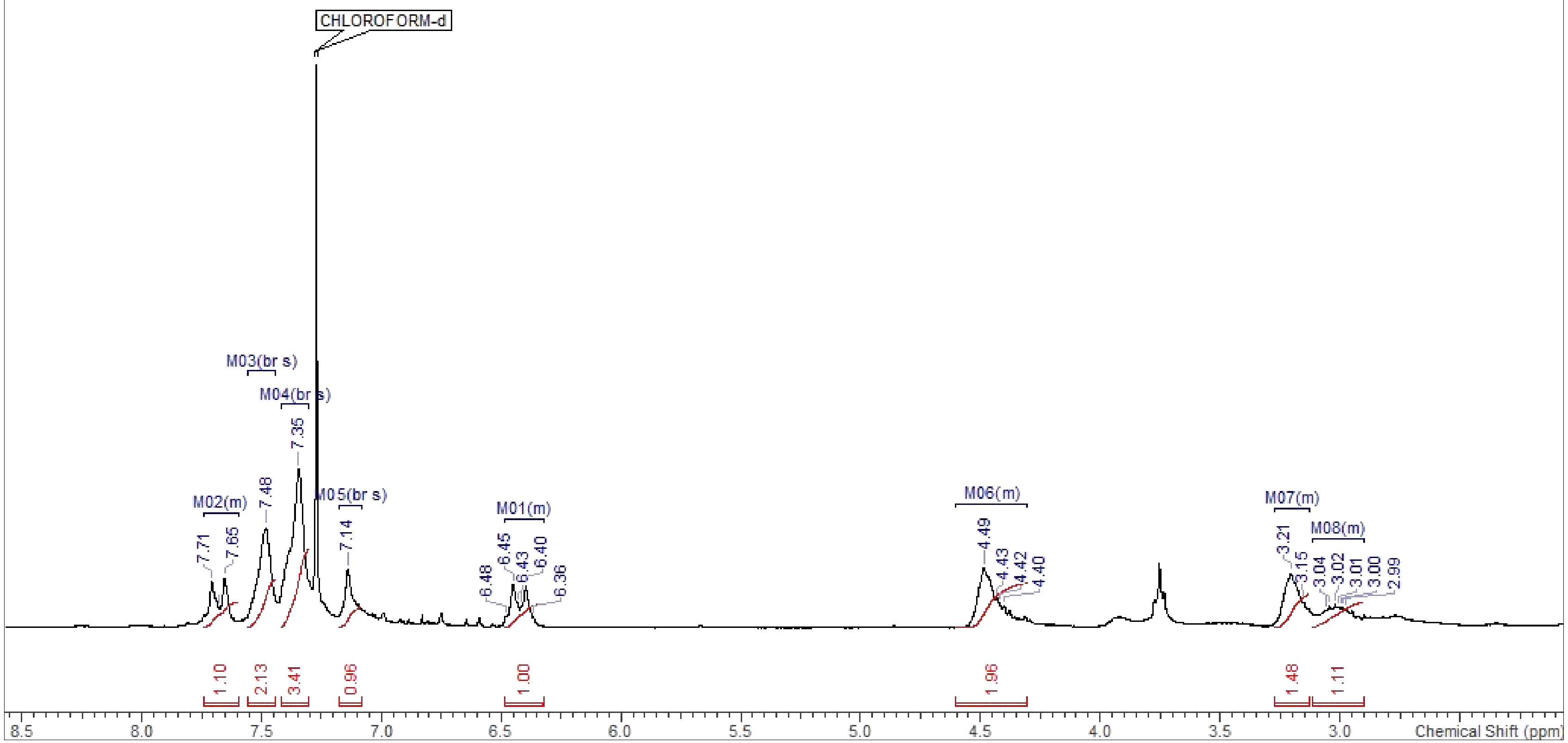

^{13}C NMR spectra of **P3ZT**

Acquisition Time (sec)	1.5897	*Comment*	NB 227b	*D*	1.5	*D1*	1.5	*DE*	18
DS	2	*Date*	10 Feb 2020 21:34:13			*Date Stamp*	10 Feb 2020 21:34:13		
File Name	E:\Bojanowski\Doktorarbeit\Analytics\NMR\c200210ubnb.227b\1\PDATA\1\1r					*Frequency (MHz)*	100.6631	*GB*	0
INSTRUM	<spect>	*LB*	1	*NS*	3200	*Nucleus*	13C	*Number of Transients*	3200
Origin	spect	*Original Points Count*	49066	*Owner*	ns	*PC*	1.4		
PROBHD	<Z130030_0004 (CPP BBO 400S1 BB-H&F-D-05 LT)>					*PULPROG*	<zgpg30>	*Points Count*	65536
Pulse Sequence	zgpg30	*Receiver Gain*	2050.00	*SF*	100.663059	*SFO1*	100.67413193649		
SI	65536	*SSB*	0	*SW(cyclical) (Hz)*	30864.20	*SWH*	30864.1975308642		
Solvent	CHLOROFORM-d			*Spectrum Offset (Hz)*	11071.9609	*Spectrum Type*	standard	*Sweep Width (Hz)*	30863.73
TD	98132	*TD0*	400	*TE*	294.9992	*Temperature (degree C)*	21.999	*UNC1*	<13C>
WDW	1								

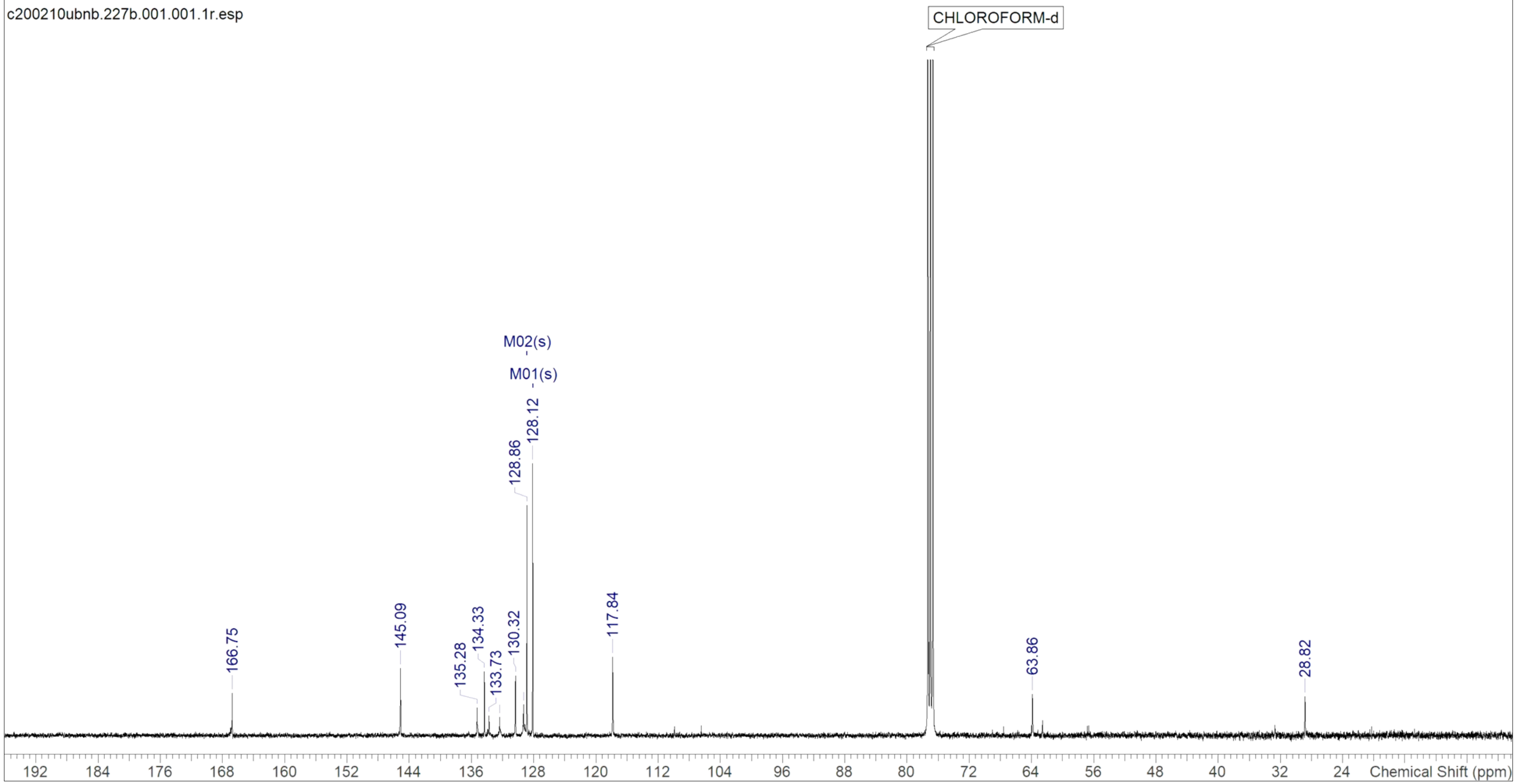

Supplement: SC-013-D2SC01867E-s001 [file SC-013-D2SC01867E-s001.pdf]
